# Evolutionary Entropy Determines Invasion Success in Emergent Epidemics

**DOI:** 10.1371/journal.pone.0012951

**Published:** 2010-09-23

**Authors:** Christopher J. Rhodes, Lloyd Demetrius

**Affiliations:** 1 Institute for Mathematical Sciences, Imperial College London, London, United Kingdom; 2 Department of Organismic and Evolutionary Biology, Harvard University, Cambridge, Massachusetts, United States of America; 3 Max Planck Institute for Molecular Genetics, Berlin, Germany; University of California, United States of America

## Abstract

**Background:**

Standard epidemiological theory claims that in structured populations competition between multiple pathogen strains is a deterministic process which is mediated by the basic reproduction number (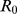) of the individual strains. A new theory based on analysis, simulation and empirical study challenges this predictor of success.

**Principal Findings:**

We show that the quantity 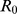 is a valid predictor in structured populations only when size is infinite. In this article we show that when population size is finite the dynamics of infection by multi-strain pathogens is a stochastic process whose outcome can be predicted by evolutionary entropy, *S*, an information theoretic measure which describes the uncertainty in the infectious age of an infected parent of a randomly chosen new infective. Evolutionary entropy characterises the demographic stability or robustness of the population of infectives. This statistical parameter determines the duration of infection and thus provides a quantitative index of the pathogenicity of a strain. Standard epidemiological theory based on 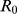 as a measure of selective advantage is the limit as the population size tends to infinity of the entropic selection theory. The standard model is an approximation to the entropic selection theory whose validity increases with population size.

**Conclusion:**

An epidemiological analysis based on entropy is shown to explain empirical observations regarding the emergence of less pathogenic strains of human influenza during the antigenic drift phase. Furthermore, we exploit the entropy perspective to discuss certain epidemiological patterns of the current H1N1 swine 'flu outbreak.

## Introduction

Recent years have seen an apparent acceleration in the rate of emergence of new infectious disease pathogens in the human population [1]. Some of these have their origins in animal (wild or domesticated) reservoirs [2]–[4], and the years since 2003 have witnessed the appearance of SARS [5], [6] and swine flu [7]. The 20^th^ century saw, for example, the emergence of pandemic 'flu in 1918, 1957 and 1968 (with a limited H1N1 re-emergent outbreak in 1977) [8], avian 'flu [9] and the rise of HIV in the 1980's [10], [11]. Additionally, antibiotic-resistant pathogens have become increasingly widespread in the past decade, particularly in healthcare settings [12]. Antigenically-variable pathogens are responsible for much of the burden of communicable disease in the world today. Therefore, developing an understanding of the factors that lead to the emergence and spread of novel pathogenic agents and strains is a topic of great interest. In recent months the emergence of a swine ‘flu (H1N1 2009) with human-to-human transmission capability has re-focussed attention on this issue [7], [13]. Likewise, studies, such of those of Creanza et al. [14] who used a computational analysis of viral nucleotide and amino acid sequence data collected during seasonal 'flu epidemics show how diversity declines over the course of an epidemic. These observations underscore the role that ecological constraint play in the evolution of pathogens.

For antigenically variable pathogens it is competition between strains that is the fundamental mechanism which determines the observed patterns of disease spread and prevalence. Diseases in this category include influenza A virus, meningococcal and pneumococcal bacterial infection, malaria and dengue fever, to name but a few. The principal epidemiological characteristics of such diseases are the absence of life-long protective immunity, cross-reactive immunity between strains and the potential for future re-infection. Each strain is in competition with the others for resources. In this case the resources are susceptible hosts, and dominance goes to those variants that are able to outpace their neighbours in their ability to infect susceptibles. From a Darwinian perspective it is the “fittest” strains that will dominate. Translating the qualitative notion of fitness into quantitative terms constitutes one of the fundamental problems in evolutionary epidemiology.

Standard epidemiological theory as largely developed by Dietz [15] and Anderson and May [10] revolves around the basic reproduction number, 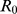, the number of secondary infectives, as the key parameter [15], [16] for analysing disease emergence, spread and vaccination strategy. In the case of structured populations, 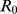 is defined as
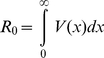
(1)where the function 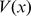 is the infectious net-reproductive function. It is an “infectious age”-dependent function that defines the rate at which an infected host generates secondary infections in the time interval following its initial infection (File S1 Section i). This theory has been extended to address competition between emergent pathogen strains using basic reproduction number as the metric for competitive dominance [16]. Selective advantage, 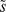, in the case of competing strains is now given by
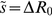
(2)where 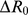 is the difference in the basic reproduction number between the incumbent and invading strain [16]. The measure of selective advantage given by equation 2 implicitly assumes that the population is infinite – a mathematical idealisation. The fact that conditions of finite size may have an effect on the outcome of selection has been recognised in a population genetics context [17] but has not been explored analytically in multi-strain epidemic models [16]. These studies, however, assumed that the populations were unstructured or demographically homogeneous. The effect of finite size in studies of selection between competing types in structured populations was first developed in Demetrius [18]. The analysis focused on demographic structure and rested on the observation that in view of the heterogeneity in structure and the finite population size,fluctuations in population numbers will occur. The ergodic theory of dynamical systems was then exploited to generate a new family of demographic variables to describe the population dynamics and its fluctuations. A diffusion process was then applied to show that the outcome of selection will now be determined by the robustness or demographic stability of the population, and regulated by the population size and certain demographic parameters which characterise the geometric properties of the infectious net-reproductive function. Robustness, the rate at which the population returns to the steady state condition after a perturbation in age-specific fecundity and mortality variables, can be formalised in terms of the statistical measure evolutionary entropy. This macroscopic variable describes the uncertainty in the age of the mother of a randomly chosen newborn. The change in basic reproduction number, the classical criterion for selective outcome, was shown to be the limit, as population size tends to infinity, of the entropic selection principle. Hence the classical models of selection are limiting cases of the entropic models. This study of competition in age-structured populations was extended by Demetrius, Gundlach and Ochs [19] to the analysis of the dynamics of selection where the heterogeneity derived from individual variations in size, metabolic condition or spatial location. The entropy parameter in this general context describes the uncertainty in the state (size, metabolic condition or spatial location) of the ancestor of a randomly chosen individual. The results of this study formed the basis of a general model of the evolutionary process which is called directionality theory.

We now apply this theory to analyse the effect of finite size in multi-strain epidemiological models where the heterogeneity derives from variability in infection age. The quantity 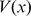 in this class of model pertains to the product of the survivorship and infectivity of infectious individuals. We will apply the formalism described in [19]–[22] to show that the invasion dynamics of competing strains in populations of finite size is predicted in terms of the macroscopic variable evolutionary entropy, *S*, which is given by
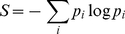
(3)and 
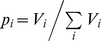
, where 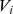 is a discretisation of the function 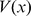.

The quantity 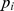 is the probability that the parent of a randomly chosen infective is in the age class i. The statistical measure, *S*, describes the uncertainty in the age of an infected parent of a randomly chosen infective.

The statistical parameter evolutionary entropy describes the rate at which the population returns to its steady state condition after a random perturbation in the age-specific fecundity and mortality variables. Entropy is analytically related to the generation time, *T* (the mean age of infection).We will use this analytical fact to show that entropy is also analytically related with the duration that the host organism is infected, and hence it can be regarded as a basic metric of pathogenicity.

Directionality theory shows that entropy *S* predicts the outcome of competition between strains. The selective advantage 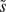 in the case of competing strains involves *S* and two additional macroscopic variables (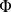 and 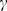, the first and third moments of a random variable defined in terms of the net-reproductive function and the probability distribution 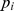). The selective advantage is now [20] given by
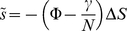
(4)Here *N* denotes the population size of infectives and 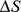 is the relative evolutionary entropy of the incumbent and the invader. The quantities 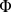 (the reproductive potential) and 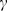 (the demographic index) are statistical parameters, and both are functions of the age-specific fecundity and survival functions which determine the infectious net-reproductive function 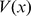.

The parameters 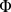 and 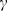 define different scenarios for the epidemiological population biology that prevail during the competitive invasion process. These quantities, in contrast to entropy, can assume positive or negative values contingent on the geometry of the infectious net-reproductive function. The demographic index, 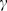, relates to the flatness or peakness of the infectious net-reproductive function 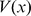: generally speaking 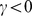 implies a peaked net-reproductive function, whereas 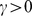 implies it is flat. This term is less influential on the dynamics as it is scaled by 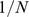, so when *N* (the number of infectives) is moderately large
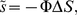
so competitive advantage is now determined by the relative entropy and the reproductive potential.

An equivalent formulation of the selective advantage, 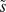, can be written in terms of the growth rate *r* and a quantity called the demographic variance, 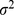 [19] , which is the second moment of a random variable defined in terms of the infectious net reproductive function and the distribution 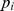,
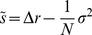
(5)where 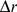 is the difference in growth rate between the incumbent and variant strains. Its equivalence to equation 4 is demonstrated in File S1, Section ii. It is evident that as 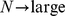 we recover equation 2, since 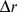 and 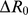 are positively correlated.

We exploit this new theory of entropic epidemiology to explain detailed empirical observations regarding the emergence of less pathogenic strains of human influenza A virus, an issue that has remained elusive when viewed through the framework of classical epidemiological theory. Moreover, we discuss the current on-going swine ‘flu (H1N1 2009) outbreak from the perspective of directionality theory, [18]–[22].

Much recent work on strain dynamics in multi-strain pathogens has focussed on the adaptations of basic epidemic models to deal with multiple strains with differing assumptions about host immune responses [23]–[26]. This has led to progress in understanding issues such as strain clustering effects, for example, but at the price of intractability when large numbers of strains are considered. By contrast, the model presented here takes a different approach, as it focuses on the emergent properties of multiple competing strains without a detailed rendering of all biological features. The penalty for generating this alternative model is that the biological detail is presented more crudely than in the more detailed epidemiological approaches. Strain selection takes place at a number of levels ranging from within hosts all the way up to the population level. In such multi-scale systems a variety of modelling approaches are needed. However, we believe that the approach presented here complements existing formulations. To make the presentation concise we will summarise certain general results of the dynamics of competition in structured populations, as elaborated in directionality theory [18]–[22], and apply them in an epidemiological context. Specifically, our model shows that in the context of emergence of new human 'flu strains in SE-Asia there will be a progressive shift to less pathogenic strains. This empirically observed pattern is consistent with our entropic perspective.

## Results

### Evolutionary Entropy and Selective Advantage

Classical epidemiological theory associates increased competitive advantage with increasing basic reproduction number, 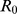 [27], [28]. The argument is that a larger 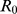 results in a faster rate of infection of susceptibles (as determined by the growth rate, *r*) thereby driving competitor pathogen strains with lower 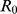 to extinction. The basic reproduction number varies for different pathogens (and their strains) and varies according to the circumstances (geographic location, age structure, population density, previous exposure etc) of the host population [10].

The claim that the outcome of competition between variant strains is a deterministic process mediated by 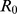 is the analogue of the claim, which goes back to Fisher [29], that the rate of increase of total population numbers – the Malthusian parameter – determines the outcome of competition between an incumbent and a variant type. These studies, in both epidemiology and population genetics assume that populations have infinite size.

Evolutionary entropy, in the context of epidemiological models, describes the uncertainty in the age of a parent of a randomly chosen infectives. This quantity is a demographic parameter that is positively correlated with the demographic stability.

Directionality theory, a study of the dynamics of competitive invasion [18]–[22], [30] of structured populations when population size is finite predicts that the outcome of selection is a stochastic process determined by evolutionary entropy and contingent on population size and two other demographic variables which characterise the geometry of the infectious net-reproductive function. Evolutionary entropy in this more general context describes the uncertainty in the state of the ancestor of randomly chosen infective. (File S1, Section i).

In this paper we exploit this general tenet to show that in finite populations with heterogeneity in age of infection the outcome of strain competition is a stochastic event determined by evolutionary entropy and contingent on the demographic parameters 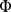 and 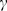. Selective advantage in this case is given by equation 4.

### Evolutionary Entropy and the Duration of Infection

One of the most significant parameters in epidemiology is the duration of host infection, *D*, which is taken to represent the period of time for which an infective is capable of transmission to susceptibles. This parameter is related to *T*, the generation time. *D* can be generally expressed in the form 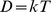 where *k* is a parameter dependent on the strain, but the characterisation of *D* will depend on the model system under consideration. In a basic *S-I-R* model, for example, the fecundity function is a constant independent of infectious age. Consequently the generation time will depend uniquely on the mortality rate (i.e. the rate of recovery from the pathogen). The generation time *T* will be inversely related to the rate at which individuals leave the infected class, denoted by 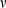. Hence for this class of models 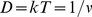. In the model described in this paper, survivorship and fecundity are functions of age, so the generation time involves both survivorship and fecundity components. The generation time *T* can be expressed in terms of the entropy function *S*. As shown by Demetrius et al. [31], the evolutionary entropy can be shown to be analytically related to *T* by
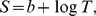
(where *b* is a strain dependent parameter) so 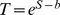. In view of the relation between *D* and *T* noted above, we have
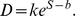
This equation asserts, therefore, that in systems with demographic heterogeneity the duration of infection will now be regulated by the pathogen entropy, *S*. This important fact underscores the fact that demographic heterogeneity dictated by 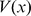 will induce significant changes in the epidemiological dynamics.

These results have particular significance in understanding the epidemiology of influenza A virus.

### Application to the Epidemiology of Influenza A

Influenza A epidemics in humans are characterised by infrequent (typically on a decadal timescale), but nevertheless significant, genetic re-assortments that lead to pandemics of new sub-types (antigenic shift) as well as within-sub-type evolution (antigenic drift) on an annual timescale. In the antigenic shift scenario it is assumed that there is very little or no host cross-immunity with previous virus types, whereas within-sub-type drift can generate strains with varying degrees of host immune response. This difference is important in determining how variants invade host populations.

Antigenic shift and antigenic drift can be characterised in terms of the demographic parameter 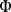. This variable is analytically related to the population growth rate, *r*, and the entropy rate *S/T* by the identity
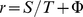
(where *T* is the generation time - see File S1 section i for parameter definitions). From this equation it follows that when 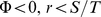.


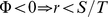
 i.e. *r* small – corresponds to a relatively small/negligible growth rate of incumbents.

But when


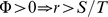
 i.e. *r* large – corresponds to a relatively large growth rate of incumbents.

We will consider these two notable epidemiological characteristics in turn:

#### Antigentic Drift: 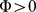


**Figure 1:**
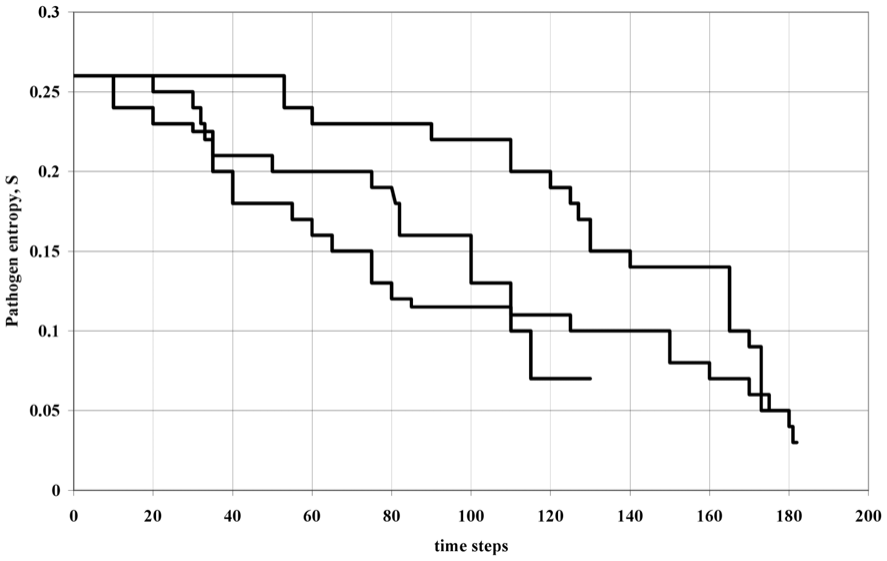
Simulation showing evolutionary entropy of dominant variant decreasing over time for three realisations of the simulation (arbitrary units). This corresponds to the scenario of 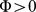, i.e. variants are competing against incumbents with positive growth rates in the host population. Because 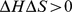, a plot of *H* versus time would yield the same pattern.

Recent work on the genetic and antigenic evolution of Influenza A H3N2 in humans has demonstrated that seasonal 'flu epidemics emerge from seed strains originating in countries of south-east Asia which subsequently spread sequentially through the global population [32]–[35]. This suggests that novel H3N2 variants compete with existing strains within the E-SE Asia circulation network with the dominant strain being responsible for generating the next global seasonal 'flu strain. Once out of the seeding region there appears to be little subsequent viral evolution [32], though as pointed out by Rambaut et al. [36] subsequent changes tend to be deleterious and so die out. When a new H3N2 variant is generated within the seeding region it competes for susceptibles with existing variants. The epidemiological picture in this densely populated SE-Asia seeding region is one of strong fluctuations of multiple circulating strains [32]. Much of this fluctuation is driven by the cross-reactive immunity and heterogeneity of host population immune response to the different strains and finite size population effects. The dynamics are characterised by repeated boom-bust cycles in the strain populations, so competition is taking place between a new variant and a rapidly growing incumbent pathogen population that has a large positive growth rate *r*, implying that the reproductive potential 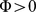. Information on the infectious net-reproductive function from which we would infer the value of 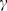 is less readily available [33]. It is plausible to assume, however, that the rate at which secondary infections are produced is broadly correlated with pathogen burden. This results in a net-reproductive function for influenza [33, Figure 1] that is slightly skewed towards the early stages of host infection, implying that 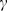 is small and negative. From Table S1, the constraints 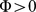 and 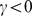 suggest that new variants will enjoy a selective advantage if their evolutionary entropy is lower than other competing strains (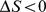) during the competitive growth phase. In this model the invasion success of the new strain will be dependent on the relative evolutionary entropy of the variant contingent on the demographic variables 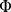 and 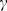. The information given in Table S1 can be invoked to determine the selective outcome of competing strains. This information underscores the difference between the deterministic process which defines the classical models and the stochastic process which characterises entropic epidemiology. A variant strain has epidemic potential and will dominate existing H3N2 strains providing that 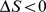. By contrast, in a deterministic model (equation 2) the outcome of competition between strains is a deterministic process predicted wholly by 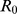 - the strain with the largest basic reproduction number will always dominate. In the entropic model there is a probability of a downward drift in the evolutionary entropy, *S*, of the dominant strain. Within the *classical* framework (infinite population size) the outcome is deterministically ordained, that is, we expect that those variants which produce more secondary infectives to dominate all others. By contrast, in a finite population (and contingent on the values of 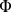, and to a lesser extent 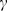) the outcome has an intrinsic stochastic component: it is the evolutionary entropy that determines success so there may be dominant variants which produce fewer secondary infectives (i.e. have a lower growth rate and 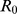) than their co-circulating competitors.

The analytic relation between evolutionary entropy and the duration of infection described above entails that lower entropy strains have a shorter duration of infection. Consequently, we expect new annual 'flu strains to have variable 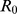 and short infectious duration relative to strains circulating in the SE-Asia seed network because of their smaller evolutionary entropy, *S*. This pattern is also seen in Table 1 where successful emergent epidemics have variable 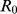 but short infectious durations. Additionally, these lower entropy strains will have a greater resilience in maintaining the chain of infection as the pathogen spreads because they have a selective advantage with respect to other strains with which they have to compete for susceptibles. Basic epidemic models [10] correlate the basic reproduction number 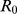 with the duration of infection, *D* which implies that short duration infections will have smaller basic reproduction numbers and would be more likely to die out. By contrast, the analysis here suggests that in the context of emergent epidemics it is the minimisation of the evolutionary entropy (which is proportional to *D* – since 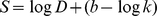) that is the determinant of emergent epidemic success, Figure 1 shows the change in entropy over time for a simulation of the invasion process by variants, and it is clear that there is a tendency to decreasing entropy over time for each run. Classical epidemic models define the proportion of the population that need to be vaccinated to eliminate a disease in terms of 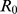, but the theory presented here shows that this is only true in the limit of infinite population which indicates that in future new vaccination criteria will be required for emergent epidemics which take into account finite population size, variability in infection profile and stochasticity effects.

**Table 1:** Basic reproduction numbers and infectious durations for a number of communicable diseases.

	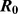	Infectious duration, *D*
Swine flu 2009	1.2 to 1.6	3 days
Seasonal flu	1.2	3–6 days
1918 flu	∼2 (up to 20)	4 days
SARS	∼3	∼9 days
Measles	>10	>8 days
Chickenpox	>7	>7 days
Mumps	>10	>12 days

Swine 'flu from Fraser et al. [13], seasonal 'flu from Earn et al. [37], 1918 'flu from Mills et al. [38], SARS from Lipsitch et al. [6], measles chickenpox and mumps from Anderson and May [10].

#### Antigenic Shift: 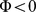


On longer timescales completely new influenza A virus sub-types occasionally emerge. These events are unpredictable and usually result in global pandemics with significantly elevated levels of 'flu-related mortality. These new sub-types are thought to arise from hybridisation of human 'flu viruses with those circulating in pigs and/or poultry [8]. Because the new virus is generated by a complete change in the HA antigenic subunit on the surface of the virus the entire global population is essentially susceptible to the disease thereby generating a pandemic. The last major antigenic shift event was in 1968 and it generated the H3N2 (Hong Kong ‘flu) strain that has been in circulation since.

Following antigenic shift the new influenza variant is in competition with an incumbent strain that is already at equilibrium in the population which suggests that the growth rate *r* is small, so 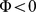. From the perspective of directionality theory (Table S1) the favourable condition for establishment of the new type is higher entropy relative to the existing circulating sub-types (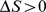), i.e. a longer duration of infection. In the antigenic shift case we are addressing infection in the entire global population so, in effect the population of infectives, 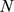 is very large. Therefore the role of the demographic index term 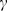 (though again it will be small and <0, for influenza) is minimal.

**Figure 2:**
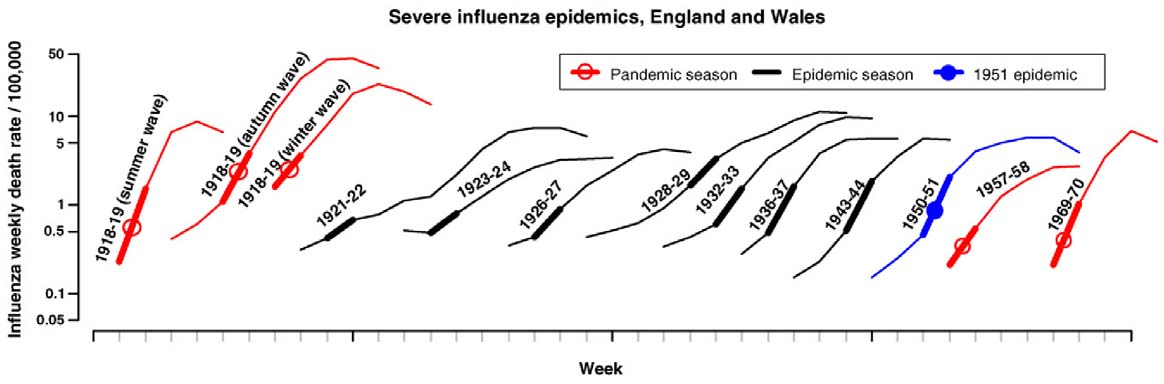
Influenza A (H1N1) weekly mortality rates in large cities in England and Wales for the major influenza outbreaks 1918 to 1951. Note the log scale on the mortality rates. The thick bars on each of the outbreaks proportional to the basic reproduction number of the epidemic.

The larger entropy of the variant corresponds to longer infectious duration than the circulating incumbents. However, the mechanism of antigenic drift (as described above) then begins to operate on this newly established sub-type and so there will be a gradual decrease in infectious duration of the dominant circulating variant with time. Given that the new virus is likely to be a hybrid animal-human type, it is likely that it is less well adapted to human hosts so it might have a low 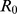. If, rather crudely, we associate infectious duration in the host with pathogenicity, directionality theory implies high pathogenicity immediately following establishment of a new sub-type followed by decreasing pathogenicity in subsequent years. That is, the new Influenza A sub-type (such as 1918, 1957 and 1968) appears to be highly pathogenic in the immediate interval following establishment, but there is a contribution to the decrease in 'flu mortality in the era following the antigenic shift by the action of competitive selection of lower entropy variants during the antigenic drift phase in the SE-Asia seeding region. When a new Influenza A virus sub-type is generated due to antigenic shift the cycle is repeated again. Clearly, there will be a decrease in the absolute influenza mortality figure (number of fatalities) during the drift phase because as time passes the overall population immunity level increases. However, the model suggests that the case fatality rate (CFR - number of fatalities per infected individuals) will decline during the drift phase due to declining pathogenicity. A decline in the CFR is observed empirically when comparing a pandemic attack year and the next subsequent epidemic [39] but there does not appear to be any detailed epidemiological analysis of long-term CFR trends during the drift phase. A recent detailed study of the epidemiology of Influenza A H1N1 in the era 1918–1951 [40] shows, Figure 2, that during the H1N1 era there is evidence of a sustained decrease in mortality rate between 1918 (autumn wave) and 1924 and likewise between 1928 and 1944. Whilst some of this progressive weakening of the influenza epidemics is due to increasing host population immunity it is likely that there is also a contribution to declining pathogenicity resulting from the mechanisms proposed in the directionality theory.

#### H1N1 (2009) Swine flu

In March 2009 the first reports of an epidemic of a novel influenza-like pathogen emerged in Mexico [13]. Analysis showed that the infectious agent in this on-going epidemic to be Influenza A H1N1 (swine flu) [7]. H1N1 has generated epidemics in humans in the past and was responsible for the 1918 influenza pandemic and a 1977 'flu epidemic. It is possible to explain the relentless spread of this current outbreak in directionality theory. This new variant has emerged from a 'flu type associated with pigs, but now has human-to-human transmission capability. This successful variant has a shorter infectious duration than other influenza A strains [13] which suggests that it may have emerged with a competitive advantage founded on its lower entropy 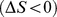 relative to other currently circulating 'flu strains. The extent of pre-existing immunity to H1N1 (swine flu) is currently unknown, but our results suggest that in humans there may be some pre-existing protection from previous exposure to influenza virus. This possibility is also noted by Fraser et al. [13]. Alternatively it may simply be a novel pandemic strain with short infectious duration. SARS had a higher 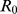 and longer infectious duration (Table 1) than swine flu yet did not have the same global impact, which reinforces the generic suggestion from entropic considerations that it is those emergent diseases with shorter infectious durations that appear to have the greater pandemic potential.

#### Existing endemic infections

**Figure 3:**
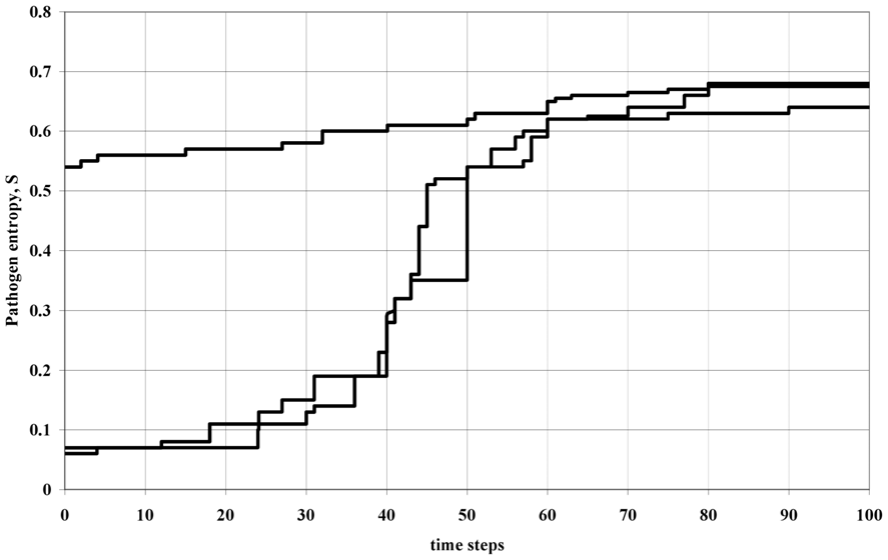
Simulations showing evolutionary entropy increasing over time for three realisations of the simulation (arbitrary units). This corresponds to the scenario of 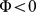, i.e. variants are competing against incumbents at equilibrium in the host population. Because 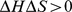, a plot of *H* versus time would yield the same pattern.

We noted above that many antigenically stable infectious diseases (measles, chickenpox for example) have comparatively long infectious durations compared with emergent infections (Table 1). Directionality theory suggests that this might be the consequence of a long evolutionary adaptation to humans by occasional mutations resulting in ever higher evolutionary entropy *S*. In this picture, for a mutation to dominate an *established* equilibrium incumbent strain it is necessary for the variant to have 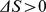, so on evolutionary time scales we see an upward drift in entropy and, consequently, ever increasing duration of infectiousness. Although such diseases do have a seasonal component to their incidence they nevertheless exist at some stable mean prevalence within the host population. The next dominant strain measles has to compete against a long-established incumbent strain that is at overall equilibrium in the population. Figure 3 shows the change (upward drift) in entropy of the most frequent variants in the population using a simulation of the invasion process. In this picture the dynamics of invasion is considered at a global scale (number of infectives 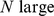) so the conventional Malthusian picture re-asserts itself. Consequently in this situation there will be proportionality between the duration of infection and 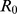, so the concept of a basic reproduction number retains its conventional role as a measure of selective advantage and, hence, its usefulness as a metric for the amount of vaccination required to eliminate a given pathogen strain. It is apparent that within the approach presented here the epidemiological context (emergent pathogen versus established equilibrium) in which the competition takes place does matter to the outcome and the properties of strains that will dominate.

## Discussion

In summary, directionality theory shows that during the fluctuating (opportunistic) competitive growth phase of multi-strain pathogen epidemic establishment it is short infectious duration (low entropy) strains that are favoured over longer infectious duration (higher entropy) strains. Moreover, they will be resilient to competition from other strains thereby giving them pandemic potential. By contrast, in established (equilibrium) populations it is longer infectious duration (high entropy) strains that have competitive advantage. This suggests that the epidemiological circumstances, opportunistic or equilibrium, that are prevalent in a host population during competitive emergence are critical in determining the properties of the dominant pathogen strain. To be clear, the stochasticity and fluctuations present in this model arise from consideration of an infection process in a finite population that has infection demographics defined by the function 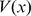. In this case the straightforward application of the concept of the basic reproductive number can be of limited usefulness as a key determinant of epidemic dynamics as there is no longer an automatic correlation between 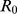 and competitive dominance.

The model presented above has some explanatory power beyond that of conventional theory in that it suggests that for Influenza A new pandemic variants generated by antigenic shift will be more pathogenic (assuming pathogenicity correlates with infectious duration) than the subsequent seasonal strains generated by the process of evolutionary antigenic drift. From a public health perspective these results suggests that monitoring of those emergent strains with a shorter infectious duration is a better indicator of pandemic risk than focussing on just 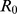, as they present an elevated threat of triggering pandemics and may need to be the target of timely vaccine development. Moreover, these results suggest the calculations of 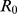 may not provide a reliable guide to the vaccination effort required to eliminate an emergent pandemic strain. The limitation of a singular focus on 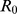 has been highlighted by Meyers [41] in the context of epidemics on networks. However, further work is needed to develop the application of directionality theory to empirical epidemiological questions such as determining the optimal vaccination coverage. Table 2 contrasts the classical and entropic models in order to emphasize their fundamental differences in explanatory and predictive power.

**Table 2:** Contrasting properties of conventional and entropic epidemic models.

Properties	Classical Model	Entropic Model
Organising parameter	Basic reproduction number 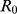	Entropy *S*
Selective advantage	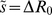	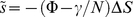
Duration of infection *D*	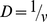	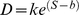
Nature of infective process	Deterministic	Stochastic

The reasons why some flu strains are more pathogenic than others is a complex issue involving specific details of host-virus interactions, but the model we propose has the attraction of capturing (on a simple criterion of pathogenicity, at least) evolution to generally less-pathogenic strains. The determinants that drive empirically observed patterns of emergence and spread of novel infectious pathogens are incompletely understood, turning as it does on the interplay of epidemiological, immunological and genetic considerations. No single model is able to capture the full complexity of this reality, but the work presented here is intended to shed some light on the criteria for invasion success and subsequent evolution of emergent strains.

Our results show that conditions of demographics of the infection process, finite population size and consideration of the prevailing epidemiological dynamics against which strain competition occurs together impose limitations on the explanatory and predictive power of any analysis based solely on the basic reproduction number. The concept of evolutionary entropy provides a framework that is stochastic in its foundation for resolving these limitations.

## Materials and Methods

### Details of the simulations

The population of infectives is divided into a number of discrete “age” classes. Each day every individual either moves up to the next age class or moves to the Recovered class with probability 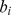. An infective in age-class *i* produces on average 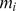 infectives. These new infectives each begin their journey through the infective stage in age-class 1. Consequently, there are two functions, defined by 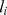 and 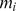, (and hence 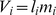 see File S1 section i) that characterise a pathogen strain and its behaviour in the host.

Transitions in the simulation are decided by a stochastic process. The simulation starts with *N* wild-type infectives. Each day each infective has its infectious age increased by 1. A random number in the interval (0,1) is then generated for each infective. If this number is <current recovery probability 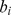 then the individual recovers, otherwise it generates its quota of secondary infectives 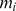. New infectives begin with infectious age = 0. For each new infective a random number in the range (0,1) is generated. If it is <mutation rate then this new infective will be a variant strain. Mutations are generated by a small perturbation to the function 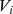 (see below “Mutation and Competition”). Each day the number of each strain is calculated so that the dominant (highest frequency) strain can be identified. The entropy, *S*, of this strain is then calculated using the demographic parameters.

To simulate the 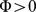 scenario in Figure 1 (which corresponds to antigenic drift in the SE Asia region) requires rapid strain growth rates (*r* large). This is done by initially allowing the total population (of all strains) to grow rapidly. Once the supply of susceptibles becomes depleted the population collapses abruptly (resource availability variable). The supply of susceptibles is then re-instated and the boom-bust cycle repeats itself. New strain variants are generated through the cycle. The purpose of this is to mimic the conditions for emergence of new variants when there is competition and growth. In this scenario variants that compete against each other are not at equilibrium in the population so we are addressing a localised competitive situation. The total population size used for this simulation was 10,000 individuals with 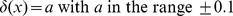 with a mutation rate of 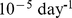.

To simulate the 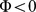 scenario in Figure 3 the supply of susceptibles is controlled to maintain the total population of infectives at a broadly constant level (i.e. resource availability constant). This reflects low-to-minimal growth rate (equilibrium, *r* small) of the incumbent. The number of infectives fluctuates around an equilibrium level and new variants attempt to invade the system whilst it is in this configuration. In this scenario the incumbent is already established at equilibrium, so we are addressing a global competitive situation. These simulations were run for 200,000 days with 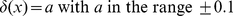 and with a mutation rate of 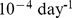.

### Mutation and competition

Each variant is characterised by the net fecundity function 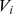. We assume that mutants are defined by 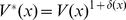 where 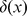 is monotonic in *x*. As a consequence, mutants arise from translation on the function 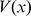 (corresponding to a change in the age of infector) or from a re-scaling of 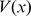 (corresponding to an increase or decrease in the net fecundity function). Monotonicity is imposed to preclude net-fecundity profiles that are large at early and late stages and low at intermediate stages.

The genotypes of mutant and wild-types are constructed so as to have positive growth rates. Consecutive time-steps of evolution are simulated by generating random numbers to decide which individuals recover or continue as infectives. To simulate competition between strains some additional growth constraints have to be applied to each scenario. In the 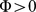 case the initial population is allowed to grow rapidly from an initial starting number 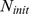. Following exhaustion of the supply of susceptibles (i.e. resources are depleted) an extrinsic mortality 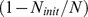 is applied to all individuals to reduce the population of infectives back down to its starting value. This process is repeated over many time-steps. In the 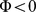 case if the total population size (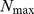) of infectives is exceeded an extrinsic mortality 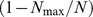 is applied probabilistically so that the population is maintained at a level that fluctuates around 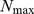.

In both scenarios, at each time step, the dominant (most frequent) genotype is determined and its entropy calculated from equation S4.The value of this entropy is recorded for the duration of the simulation. It should be noted that these simulations are not based on an elaboration of *S-I-R* models of the usual type where susceptibles and infectives interact via conventional mass-action terms. Here, the population of infectives is directly manipulated to reflect the kind of epidemiological dynamics that are typically seen in the emergent and equilibrium phases [42].

## Supporting Information

Table S1 Invasion criteria in the entropy model. *“a.s. = Almost surely” refers to the fact the result is a stochastic process. The criteria for large and small population size are defined in more detail in Demetrius et al. [19]. The criteria noted in Table S1 have been tested against simulation where they have been shown to be replicated.
(0.07 MB DOC)

Figure S1 Life cycle for an infective corresponding to the matrix in equation S1 with 4 infectious age classes.
(0.06 MB TIF)

File S1 Supporting text.
(0.22 MB DOC)
