# Supplementary material for: Evolutionary Entropy Determines Invasion Success in Emergent Epidemics
Source: PLoS One. 2010 Sep 23;5(9):e12951. doi: 10.1371/journal.pone.0012951 (PMC2944876; doi:10.1371/journal.pone.0012951)
Supplement: Table S1 — Invasion criteria in the entropy model. *“a.s. = Almost surely” refers to the fact the result is a stochastic process. The criteria for large and small population size are defined in more detail in Demetrius et al. [19]. The criteria noted in Table S1 have been tested against simulation where they have been shown to be replicated. (0.07 MB DOC) [file pone.0012951.s001.doc]

**Invasion Criteria**

Measure of selective advantage $\widetilde{s}=-\left(\Phi-\gamma/N\right)\Delta S$

| **Ecological Constraints** | **Invasion Condition** | **Selective Outcome for the Variant** |
| --- | --- | --- |
| $\Phi<0,\gamma>0$ | $\Delta S>0$ $\Delta S<0$ | Invasion a.s.  Extinction a.s. |
| $\Phi>0,\gamma<0$ | $\Delta S<0$ $\Delta S>0$ | Invasion a.s.  Extinction a.s. |
| $\Phi<0,\gamma<0$ Large population size  Small population size | $\Delta S>0$ $\Delta S<0$ $\Delta S>0$ $\Delta S<0$ | Invasion a.s.  Extinction a.s.  Invasion with prob. increasing with *M*.  Extinction with prob. increasing with *M*. |
| $\Phi>0,\gamma>0$ Large population size  Small population size | $\Delta S<0$ $\Delta S>0$ $\Delta S<0$ $\Delta S>0$ | Invasion a.s.  Extinction a.s.  Invasion with prob. decreasing with *N*.  Extinction with prob. increasing with *N*. |
