# Supplementary figures and images for: Evolutionary Entropy Determines Invasion Success in Emergent Epidemics
Source: PLoS One. 2010 Sep 23;5(9):e12951. doi: 10.1371/journal.pone.0012951 (PMC2944876; doi:10.1371/journal.pone.0012951)

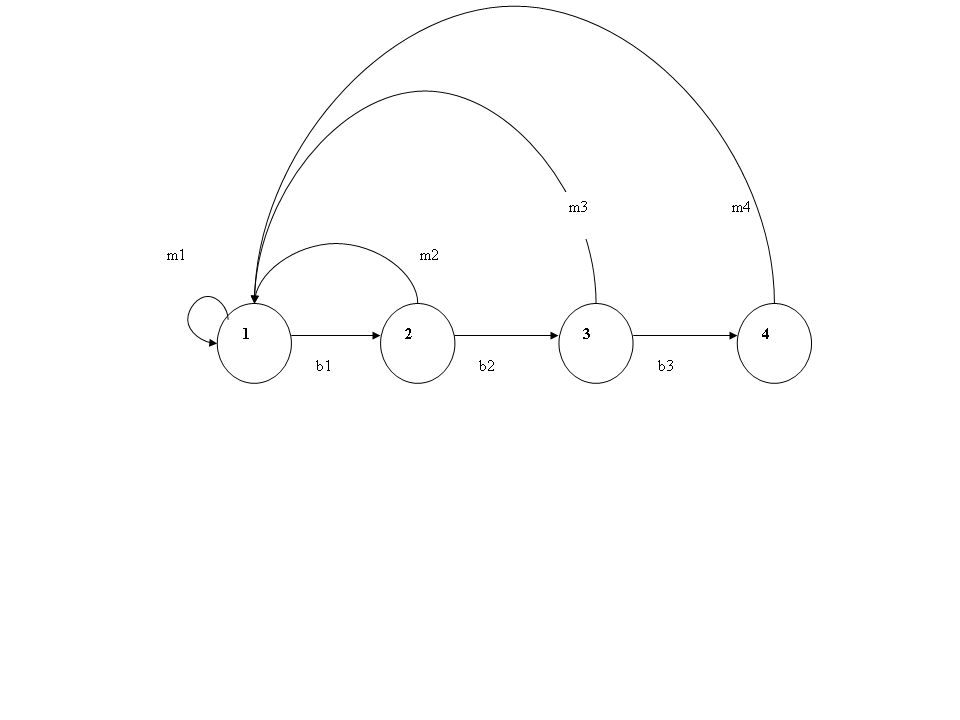

Supplement: Figure S1 — Life cycle for an infective corresponding to the matrix in equation S1 with 4 infectious age classes. (0.06 MB TIF) [file pone.0012951.s002.tif]
