# Supplementary material for: Evolutionary Entropy Determines Invasion Success in Emergent Epidemics
Source: PLoS One. 2010 Sep 23;5(9):e12951. doi: 10.1371/journal.pone.0012951 (PMC2944876; doi:10.1371/journal.pone.0012951)
Supplement: File S1 — Supporting text. (0.22 MB DOC) [file pone.0012951.s003.doc]

**Supporting Information**

**Evolutionary entropy determines invasion success in emergent epidemics**

Christopher. J. Rhodes ^a1^ and Lloyd. Demetrius ^b^

*a. Institute for Mathematical Sciences, Imperial College London, South Kensington, London, United Kingdom.*

*b. Department of Organismic and Evolutionary Biology, Harvard University, Cambridge, USA and Max Planck Institute for Molecular Genetics, Berlin, Germany.*

^1^To whom correspondence should be addressed. Email: c.rhodes@imperial.ac.uk

**Section i)**

It is the outcome of competitive dynamics of multi-strain diseases that determines the empirically observed patterns of disease incidence for pathogens such as malaria, dengue and influenza, for example. Each strain competes with the other circulating strains for susceptible hosts, and it is the strain that can out-compete the others (i.e. the “fittest” strain) that will dominate. Translating these qualitative notions of strain “fitness” into quantitative terms that can be used in modelling has been, and remains, challenging.

In an ecological context this challenge has been resolved by directionality theory which was developed from a statistical mechanical perspective to account for the role of population size and demographic heterogeneity in determining the outcome of competitive invasions. Directionality theory revolves around the analytical and empirical fact that the competitive dynamics of invasion is a stochastic process predicted by evolutionary entropy. Entropy in this ecological context is a statistical parameter which describes the uncertainty in the age of the mother of a randomly chosen newborn. It also characterises the rate at which a population returns to steady state conditions after a perturbation – hence it defines robustness, or demographic stability.

The epidemiological models analysed in this paper have invoked the formalism of directionality theory to analyse the evolution of pathogens in populations of finite size and heterogeneous demographic structure. The central concept in these models is the evolutionary entropy of a pathogen. Our model integrates demographic variability due to multiple pathogen strains into epidemiological studies drawn from the work of Levin et al. (S1) and recent developments due to Wallinga and Lipsitch [16]. A life-cycle graph for a simple communicable disease is shown below in Figure S1.

It is assumed here that the infectives are divided into 4 age-classes, and the structuring of the model with discrete age-classes is critical to the development of the model. The “age” of an infective refers to the time elapsed since they first became infected by the pathogen. For a basic communicable disease each of these age-classes might represent the days elapsed following the infection event, in this case an infectious duration of four days. The transitions from one age-class to the next are determined by the values of $b_{i}$. In this case the $b_{i}$ reflect “survivorship” of the infected individuals (i.e. the transition to recovered class). Now, each age-class of the infectious group can generate “new” infectives that start their lives in age-class 1, hence the inclusion of the self-loop on class 1. Therefore, the $m_{i}$represent the mean age-specific generation of new infectives per infective. In an epidemic these are generated by the mixing of the infectious individuals in each age-class with susceptibles, so the $m_{i}$ will be dependent on the numbers of infectives in class *i* and the overall number of susceptibles that are available for infection. The structure of the model is shown in Figure S1.

The life-cycle matrix for the infectives is as follows:

$\left[\begin{matrix} m_{1} & m_{2} & m_{3} & m_{4} \\ b_{1} & 0 & 0 & 0 \\ 0 & b_{2} & 0 & 0 \\ 0 & 0 & b_{3} & 0 \end{matrix}\right]\left[\begin{matrix} n_{1,t} \\ n_{2,t} \\ n_{3,t} \\ n_{4,t} \end{matrix}\right]=\left[\begin{matrix} n_{1,t+1} \\ n_{2,t+1} \\ n_{3,t+1} \\ n_{4,t+1} \end{matrix}\right]$ (S1)

where the$m_{i}$ determine the age-specific generation of secondary infectives (note that this is a discrete-time model). The $n_{i}$are the numbers of infectives in each of the 4 infectious age-classes. From this model it is possible to define the following quantities:

$l_{i}=b_{1}.b_{2}....b_{i-1}$which represents survivorship to age-class *i*.

$V_{i}=l_{i}m_{i}$ which is the net production of infectives at age-class *i*

The growth rate, *r*, of the population of infectives is determined by solving Lotka’s equation for the real root

$1=\sum_{i} \frac{V_{i}}{e^{ri}}$ (S2)

The population growth rate, *r*, satisfies a variational principle analogous to the extremal principle for free energy in equilibrium thermodynamics [21, S2]. Accordingly, the growth rate can be expressed as

$r=H+\Phi$ (S3)

Where *H* is the entropy and $\Phi$is the reproductive potential. These quantities are defined by

$H=-\frac{\sum_{i} p_{i}\log p_{i}}{\sum_{i} ip_{i}}\equiv\frac{S}{T}$ (S4)

and

$\Phi=\frac{\sum_{i} p_{i}\log V_{i}}{\sum_{i} ip_{i}}\equiv\frac{E}{T}$ (S5)

where $T=\sum_{i} ip_{i}$ and $p_{i}=V_{i}/e^{ri}$.

*E* is called the net reproductive index and it describes the production of infectives averaged over all age-classes; *S* is the demographic entropy and is a measure of the uncertainty of the age of the infected “parent” of a given infective individual; *T* is the generation time, i.e. the mean age of the infector when it generates a secondary infective. From this it can be seen how the evolutionary entropy *S* of an infectious variant is correlated with the uncertainty in the age of the infector and, hence, to the duration of infection. The longer the duration of infection (i.e. the more age-classes) the greater the uncertainty in the age of the infected parent, and so the larger the evolutionary entropy. To be clear on two points i) when we refer to the “age” of an infective what is being referred to is the age-class to which it belongs, that is, the time elapsed since the individual became infected, and ii) the evolutionary entropy of a pathogen is the entropy of the population of individuals who are infected with the pathogen. By analogy with equilibrium thermodynamics, the entropy associated with spatial ordering we now have an entropy measure that is associated with temporal uncertainty in the life-cycle model of the infection.

The quantity $\Phi$(reproductive potential) governs the epidemiological dynamics that are prevalent when strain competition is taking place. Now,

$\Phi=r-H$ (S5)

The function $\Phi$can assume positive and negative values. In view of equation S5 we have:

$\Phi<0\Rightarrow r<H$and $\Phi>0\Rightarrow r>H$ (S6)

Negative reproductive potential implies that growth rates are low and an invading variant has to compete against an incumbent that is already at equilibrium in the host population. When $\Phi>0$the situation is very different. This time the variant is competing against strains that are growing in number. It is these two different epidemiological circumstances that determine the specific properties of the dominant variants.

Directionality theory predicts that the outcome of competition between variant strains is predicted by $\Delta S$ and the two demographic variables $\Phi$(described above) and $\gamma$. The parameter $\gamma$. The parameter $\gamma$, the third moment of a random variable determined by the infectious net-reproductive function, is given by:

$\gamma=2\sigma^{2}-\frac{3}{T}\sigma^{2}\left(\sum_{j} p_{j}W_{j}\right)+\frac{\sum_{j} p_{j}W_{j}^{3}}{T}$ (S9)

where$\sigma^{2}=\sum_{j} p_{j}W_{j}^{2}/T$and $W_{j}=jH_{j}+\log p_{j}$.

**Section ii)**

From Demetrius *et al.* [17] the following perturbation relations for changes in the growth rate $r$, entropy $H$, and demographic variance $\sigma^{2}$are given:

$\Delta r=\Phi\delta$ (S10)

$\Delta H=-\sigma^{2}\delta$ (S11)

$\Delta\sigma^{2}=\gamma\delta$ (S12)

From which it follows that

$\Delta r\Delta H=-\Phi\sigma^{2}\delta^{2}$ (S13)

$\Delta H\Delta\sigma^{2}=-\gamma\sigma^{2}\delta^{2}$ (S14)

Directionality theory [16] shows that the selective advantage of an invading variant over an incumbent is given by

$s=\Delta r-\frac{1}{N}\Delta\sigma^{2}$ (S15)

The equation S15 expresses *S* in terms of the growth rate *r*, and the demographic variance $\sigma^{2}$. Also the Malthusian result is recovered as$N\to\infty$. We now show that equation S15 is equivalent to equation 4. In what follows we present the argument in terms of *H*, but as $\Delta S\Delta H>0$the result holds for *S* also.

In order to establish the equivalence of equation S15 and equation 4 we write

$s=-\left(\Phi-\gamma/N\right)\Delta H$ (S16)

$\widetilde{s}=\left(\Delta r-\frac{1}{N}\Delta\sigma^{2}\right)$ (S17)

and show that $s\widetilde{s}>0$.

From equations S16 and S17 we obtain

$s\widetilde{s}=\left(\gamma/N-\Phi\right)\left(\Delta r^{2}-\frac{1}{N}\Delta\sigma^{2}\right)\Delta H$ (S18)

which can be expanded to

$s\widetilde{s}=\frac{1}{N}\left(\gamma\Delta r\Delta H+\Phi\Delta\sigma^{2}\Delta H\right)-\frac{\gamma}{N^{2}}\Delta\sigma^{2}\Delta H-\Phi\Delta r\Delta H$ (S19)

Now, using S16 and S17 this becomes

$s\widetilde{s}=\frac{1}{N}\left(-\Phi\gamma-\Phi\gamma\right)\sigma^{2}\delta^{2}+\frac{\gamma^{2}}{N^{2}}\sigma^{2}\delta^{2}+\Phi^{2}\sigma^{2}\delta^{2}$ (S20)

leading to

$s\widetilde{s}=\left(\Phi-\frac{\gamma}{N}\right)^{2}\sigma^{2}\delta^{2}$ (S21)

i.e. $s\widetilde{s}>0$. Hence the measures of selective advantage are equivalent, that is $s>0$and $\widetilde{s}>0$. Consequently, the two formulations of selective advantage predict the same outcome, though their magnitudes are related by a factor$\sigma^{2}$.

**Supporting Information References**

S1 ) Levin BR, Bull JJ, Stewart FM (1996) Mathematical Biosciences 132: 69-96.

S2) Arnold L, Demetrius L, Gundlach VM (1994) Annals of Applied Probability 4: 859-901.
